# Common Genetic Variations in the NALP3 Inflammasome Are Associated with Delayed Apoptosis of Human Neutrophils

**DOI:** 10.1371/journal.pone.0031326

**Published:** 2012-03-05

**Authors:** Robert Blomgran, Veronika Patcha Brodin, Deepti Verma, Ida Bergström, Peter Söderkvist, Christopher Sjöwall, Per Eriksson, Maria Lerm, Olle Stendahl, Eva Särndahl

**Affiliations:** 1 Division of Medical Microbiology, Department of Clinical and Experimental Medicine, Faculty of Health Sciences, Linköping University, Linköping, Sweden; 2 Division of Cell Biology, Department of Clinical and Experimental Medicine, Faculty of Health Sciences, Linköping University, Linköping, Sweden; 3 Division of Rheumatology, Department of Clinical and Experimental Medicine, Faculty of Health Sciences, Linköping University, Linköping, Sweden; 4 Division of Cardiovascular Medicine, Department of Medical and Health Sciences, Faculty of Health Sciences, Linköping University, Linköping, Sweden; 5 Department of Clinical Medicine, School of Health and Medical Sciences, Örebro University, Örebro, Sweden; 6 Center for Infectious Medicine, Karolinska Institute Huddinge, Stockholm, Sweden; University of Louisville, United States of America

## Abstract

**Background:**

Neutrophils are key-players in the innate host defense and their programmed cell death and removal are essential for efficient resolution of inflammation. These cells recognize a variety of pathogens, and the NOD-like receptors (NLRs) have been suggested as intracellular sensors of microbial components and cell injury/stress. Some NLR will upon activation form multi-protein complexes termed inflammasomes that result in IL-1β production. NLR mutations are associated with auto-inflammatory syndromes, and our previous data propose *NLRP3* (Q705K)/*CARD-8* (C10X) polymorphisms to contribute to increased risk and severity of inflammatory disease by acting as genetic susceptibility factors. These gene products are components of the NALP3 inflammasome, and approximately 6.5% of the Swedish population are heterozygote carriers of these combined gene variants. Since patients carrying the Q705K/C10X polymorphisms display leukocytosis, the aim of the present study was to find out whether the inflammatory phenotype was related to dysfunctional apoptosis and impaired clearance of neutrophils by macrophages.

**Methods and Findings:**

Patients carrying the Q705K/C10X polymorphisms displayed significantly delayed spontaneous as well as microbe-induced apoptosis compared to matched controls. Western blotting revealed increased levels and phosphorylation of Akt and Mcl-1 in the patients' neutrophils. In contrast to macrophages from healthy controls, macrophages from the patients produced lower amounts of TNF; suggesting impaired macrophage clearance response.

**Conclusions:**

The Q705K/C10X polymorphisms are associated with delayed apoptosis of neutrophils. These findings are explained by altered involvement of different regulators of apoptosis, resulting in an anti-apoptotic profile. Moreover, the macrophage response to ingestion of microbe-induced apoptotic neutrophils is altered in the patients. Taken together, the patients display impaired turnover and clearance of apoptotic neutrophils, pointing towards a dysregulated innate immune response that influences the resolution of inflammation. The future challenge is to understand how microbes affect the activation of inflammasomes, and why this interaction will develop into severe inflammatory disease in certain individuals.

## Introduction

Phagocytic cells, neutrophil granulocytes and macrophages, are important mediator cells in the early immune response to invading pathogens. These immune cells are able to recognize a variety of pathogens through cell surface and intracellular receptors, including members of the Toll-like receptor (TLR) and Nod-like receptor (NLR) families (reviewed in: [1]). Engagement of TLRs results in the activation of MAPK and NF-κB signalling pathways, leading to expression and release of pro-inflammatory cytokines and antimicrobial peptides as well as induction of cell death. Activation of intracellular NLR by a variety of microbial molecules leads to inflammasome formation, caspase-1 activation and subsequent formation and release of interleukin-1β (IL-1β), thereby creating an intracellular surveillance system for pathogens [2], [3].

NALP3 (formerly known as cryopyrin) belongs to the family of NLR proteins. Upon activation, NALP3 assembles with the adaptor protein ASC to form a protein-complex termed the NALP3 inflammasome [4]. CARD-8 (also known as TUCAN) has been suggested to be a binding partner of NALP3 but its functional role in inflammasome regulation is not clear. The assembled inflammasome activates the protease caspase-1, which then cleaves and produces the pro-inflammatory cytokines IL-1β and IL-18 from their inactive pro-forms. β-amyloid, *Staphylococcus aureus*, bacterial RNA, and uric acid crystals are some of the stimuli found to activate the NALP3 inflammasome [5], [6], [7], [8].

In addition to their role in inflammation, NLRs have also been indicated to link innate immunity to cell death signalling (reviewed in: [9]). The NLR-linked cell death involves apoptosis and necrosis but also the two newly recognised cell death programmes, pyroptosis and pyronecrosis [10], [11]. Apoptosis [12] is triggered by a wide array of intracellular and extracellular signals via two main routes, the intrinsic pathway and the extrinsic pathway, respectively (reviewed in: [13]). The intrinsic route is activated inside the cell and is mediated by mitochondrial damage and activation of caspase-9. The extrinsic route is initiated by ligation and activation of extracellular death receptors, leading to caspase-8 activation. Both caspase-9 and -8 will subsequently activate downstream caspase-3, leading to apoptosis. The apoptotic process in neutrophils is controlled by several anti-apoptotic molecules, including the Bcl-2 family protein Mcl-1 and Akt (also known as protein kinase B) [14].

During acute inflammation, neutrophil granulocytes are recruited to the site of infection to phagocytose and clear the tissue from invading pathogens. Since they are short-lived and readily go into apoptosis, tissue macrophages are recruited to ingest the apoptotic neutrophils to prevent tissue damage and facilitate resolution of inflammation (reviewed in: [15], [16], [17].

Cryopyrin-associated periodic syndromes (CAPS) are systemic auto-inflammatory diseases that appear to depend on heterozygous mutations in the gene encoding NALP3, *i.e. NLRP3* (Gen Bank: NG 007509.2) (reviewed in: [18]). CAPS-associated mutations in the *NLRP3* gene are thought to cause constitutive inflammasome assembly and thereby a constant and uncontrolled production of IL-1ß [4], [19]. Patients suffering from CAPS often show dramatic improvement upon IL-1β blockade using an IL-1β receptor antagonist (IL-Ra) [18], [20], which indicates an important role of this cytokine in the pathogenesis of these diseases. Traditionally, the CAPS include Familial Cold Auto-inflammatory Syndrome (FCAS, also known as Familial Cold Utricaria), Muckle Wells Syndrome (MWS) and Neonatal Onset Multisystem Inflammatory Disease (NOMID). Over-expression of NALP3 and CAPS-associated mutant *NLRP3* , respectively, have been shown to induce cell death in monocytes [21], [22], [23], [24].

We have previously reported on a patient with a long history of inflammatory disease resulting from excessive IL-1β production, who was found to be a heterozygous carrier of two common polymorphisms in genes encoding proteins of the NALP3 inflammasome, *i.e. NLRP3* (Q705K) and *CARD-8* (C10X) (Gen Bank: NM 001184900) [25]. The patient's phenotype was distinct from those of typical FCAS, MWS or NOMID, thereby adding to the spectrum of CAPSs.

One distinct feature of this patient was an easily triggered and prolonged inflammatory response with accumulation of neutrophil granulocytes, suggesting that the normal resolution of inflammation was impaired. We therefore investigated if the process of apoptosis and elimination of apoptotic cells were altered in four patients carrying the *NLRP3/CARD-8* polymorphisms. We could show that neutrophil apoptosis, as detected by annexin V staining and mitochondrial damage, was significantly delayed and linked to increased expression and activity of the anti-apoptotic proteins Akt and Mcl-1. Impaired neutrophil apoptosis could thus affect the normal resolution and prolong inflammation in these patients.

## Materials and Methods

### Reagents and antibodies

The chemicals used were as follows: Benzonase (Merck); luminol (5-amino-2,3-dihydro-1,4-phtalazinedione), Dimethyl sulfoxide (DMSO), fMet-Leu-Phe, phorbol 12-myristate 13-acetate (PMA), diphenylene iodinium (DPI) and purified lipopolysaccharide (LPS) from *Escherichia coli* O26:B6 from Sigma Chemical Co; Annexin V apoptosis kit (R&D Systems); Polymorphprep™ and Lymphoprep™ (Axis shield PoC AS). The reagents used for cell culture *i.e.* RPMI-1640, Dulbecco's Modified Eagle's Medium (DMEM), L-glutamine, fetal calf serum, came from Life Technologies. Recombinant human gamma-Interferon (IFN-γ), AKT inhibitor 10-(4′-(N-diethylamino)butyl)-2-chlorophenoxazine (AKT-X), ERK inhibitor PD 98059, and the caspase-1 inhibitor z-YVAD-fmk were from Calbiochem.

The primary antibodies and their sources were as follows: Rabbit polyclonal antibodies against Mcl-1 (sc-819), and β-actin (sc-1616) were purchased from Santa Cruz; antibodies directed towards phosho-Akt (Ser473; 193H12; rabbit mAb), total-Akt (9272; rabbit polyclonal), and rabbit mAb against phospho-p44/42 MAPK (Thr202/Tyr204; 197G2) and total-p44/42 MAPK (Erk1/2; 137F5) from Cell Signalling Technologies. The fluorescein isothiocyanate (FITC)-conjugated antibody recognising the ligand-binding domain of the CD11b-integrin (mouse mAb, CBRM1/5) was from eBioscience. Antibodies (mAb) directed against CD11b (P3) or CD18 (P1–2, P4), their isotypic antibodies, and the FITC-conjugated secondary antibodies were from Dako.

### Study subjects

Blood was collected from four patients of Northern European descent (3 men and 1 woman; mean age: 59.2 (33–66) years old). The patients were all admitted to the clinic due to inflammatory related diseases without specified diagnosis. They all displayed high CRP and WBC with neutrophil granulocytes predominating, but their symptoms varied and included recurrent fever (P1, 3–4), arthritis (P1–3) and myalgia (P3–4). (For more detailed Case reports, see Data S1.) Genetic analysis showed that the patients carried the *NLRP3* (Q705K; rs35829419) and *CARD-8* (C10X; rs2043211) polymorphisms [25], but did not carry any disease-causing mutations in the *NLRP3*, *MEFV* or *TRAPS* genes. Healthy volunteers, matched in age and gender of the patients with normal routine laboratory tests (Table 1) and wild type *NLRP3* gene, were used as controls in a paired patient-control setting for the *in vitro* studies (denoted controls). Two controls carried the common *CARD-8* (C10X) polymorphism, which allele frequency is found in 34% of the Swedish population [25]. In addition, coded blood donor blood from 3 healthy asymptomatic carriers of the *NLRP3* (Q705K) and *CARD-8* (C10X) polymorphisms and from 6 healthy non-carriers was collected (denoted carriers and non-carriers, respectively), as well as blood from randomly chosen healthy blood donors (denoted donors).

**Table 1:** Laboratory characteristics of patients and controls.

	P1 (*1977) ♂	P2 (*1952) ♂	P3 (*1970) ♀	P4 (*1944) ♂	C1-4^b^	*p* values^c^
	Q705K/C10X	Q705K/C10X	Q705K/C10X	Q705K/C10X^a^		
**CRP** (mg/L)^d^	129	67	23	82	<10±0	0.0187
normal range <10						
**ESR** (mm/h)^d^	80	64	8	56	5.2±3.2	0.0239
normal range 1–12						
**WBC** (×10^9^/L)^d^	23.3	11.6	12.2	15.7	5.6±0.8	0.0098
normal range 3.5–8.8						
**IL-1ß** (pg/mL)						
Plasma	1.6	1.5	-	1.6	0.4±0.13	<0.0001
Supernatant	99	137	69	55	28±11	0.0172
LPS+ATP	441	837	431	515	536±142	ns^e^
**CD18** (MFI)	239	329	210	198	257±123	ns
**CD11b** high affinity (MFI)	80	116	50	27	84±46	ns
**ROS** (peak value, CPM)						
fMet-Leu-Phe (×10^7^)	35.8	-	7.8	4.7	5.1±2.5	ns
PMA (×10^8^)	4.7	-	2.4	4.8	3.3±1.3	ns

* Year of birth.

a Homozygote carrier of *CARD-8* (C10X).

b Data are given as mean ± SD,

c Student's t-test was used to compare P1-P4 against their age and gender (♂, male; ♀, female) matched controls (C1–C4).

d Measured at the accredited Clinical Chemistry Laboratory at Linköping University Hospital, Sweden.

e ns = not statistically significant.

At the time of experiments, P1 and P4 were treated with the IL-Ra anakinra (Kineret™; 100 mg subcutaneously every 24 h), and due to the ongoing treatment, the patients had no clinical symptoms at the time of experiment. According to the manufacturer, the half-life of this drug is 4–6 h, and administration of anakinra was therefore withdrawn from the patients for at least 24 h prior to performance of experiments (unless otherwise stated). P2 started treatment with anakinra after the experiments were performed and his symptoms improved. P3 was on a steady maintenance dose of colchicine and low-dose prednisolone, and her disease has, so far, not required additional therapy.

### Preparation of human neutrophils and macrophages

Peripheral human blood was drawn from patients and healthy volunteers and collected in heparin-containing vacutainer tubes, and neutrophils and monocytes were isolated by means of density gradient centrifugation, as previously described [26], [27]. Briefly, whole blood was layered over a gradient consisting of Lymphoprep™ layered over Polymorphprep™, and centrifuged for 40 min at 480× *g* at room temperature. The band rich in polymorphonuclear cells was collected and contaminating erythrocytes were removed by hypotonic lysis. The obtained cell suspension, consisting of >95% neutrophils, was washed twice and resuspended in RPMI-1640 supplemented with 10% heat inactivated fetal calf serum and 2 mM L-glutamine (referred to as RPMI-medium). The cells were kept on ice prior to the experiments. Macrophages were, similarly isolated using gradient centrifugations, prepared from peripheral blood monocytes that had been cultured in DMEM containing 10% human serum for 6–8 days. The medium was changed every 3 days and was replaced with DMEM without serum before being used. As indicated in the figure legends, some experiments did not include all four patients, since in some matched control individuals the number of isolated neutrophils was too few to be able to complete all analyses.

### Bacteria

*Staphylococcus aureus* strain Wood 46 (American Type culture Collection) was grown statically overnight at 37°C, diluted and cultured under agitation for an additional 3 h to obtain bacteria in log phase [28]. The bacteria were washed, resuspended in RPMI-medium and kept on ice until further handling.

### Induction of neutrophil apoptosis

Freshly isolated neutrophils (2×10^6^) were seeded in a 24-well tissue culture plate, after which they were pre-warmed for 10–15 min at 37°C in a humidified CO_2_ incubator (5% CO_2_, 95% air) and subsequently exposed to pre-warmed bacteria (bacteria:cell ratio of 10∶1) or medium (spontaneous apoptosis) [27], [28]. Infection was stopped after 1 h by adding gentamicin (50 µg/ml), and the incubation was continued for up to 20 h at 37°C in 5% CO_2_. At indicated time points, the cells were washed with phosphate buffered saline (PBS) and used for further analysis.

### Measurement of apoptosis

As an indicator of early apoptosis, the exposure of phosphatidylserine (PS) on the surface of neutrophils was analyzed by using FITC-conjugated annexin V/propidium iodide (PI)-counter staining (R&D Systems), as previously described [29]. The fluorescence (FL-1 versus FL-2) of individual cells was determined by flow cytometry using a FACSCalibur (Becton Dickinson, San Jose, CA/USA). At least 10,000 cells were counted in each sample and analyzed using Cell Quest software. Gating using forward scatter (FSC) versus side scatter (SSC) was performed to exclude cell debris. A rise in FITC fluorescence (FITC+/PI-), corresponding to increased exposure of PS, was considered to reveal early apoptotic neutrophils, whereas elevation of both FITC and PI -fluorescence was regarded as indicating late apoptotic or necrotic cells [30].

### Mitochondrial membrane potential (ΔΨm)

Mitochondrial membrane potential (ΔΨm) was assessed by use of tetramethylrhodamine ethyl ester (TMRE), which accumulates in the mitochondrial matrix. Decreased ΔΨm was indicated by a reduction in the intensity of TMRE-induced red fluorescence as detected at the end of experiments by flow cytometric analysis [27]. Briefly, neutrophils were collected and loaded with RPMI-medium supplemented with 1 µM TMRE; this was done for 15 min in the dark at 37°C. Thereafter, the cells were analysed by performing flow cytometry (FACSCalibur), using an activating wavelength of 488 nm, and monitoring the red fluorescence in the FL-3 channel (650 LP filter). At least 10,000 cells were counted in each sample and analyzed using Cell Quest software. Gating using FSC versus SSC was performed to exclude cell debris.

### Protein detection by Western Blot

Protein detection by western blotting was performed as previously described [28]. In brief: neutrophils (2×10^6^ per sample) were incubated at 37°C. Cells were withdrawn at different time points, given ice-cold PBS supplemented with 1 mM Na_3_VO_4_ and 1 mM Pefabloc, and then subjected to rapid centrifugation. Each pellet was dissolved in Laemmli sample buffer and boiled for 10 min. Equal amounts of proteins were separated by 8–16% SDS polyacrylamide gel electrophoresis (PAGE) and electrotransferred onto nitrocellulose membranes. The membranes were blocked with 5% milk powder in PBS Tween-20 (0.075%) and incubated with primary antibodies recognizing Mcl-1 (1∶250), AKT (1∶500), phospho-AKT (1∶1000), total-ERK and phospho-ERK (1∶1000), or with β-actin (1∶2000) as a loading control. If the molecular weight of the protein of interest overlapped with the weight of another protein, the blots were stripped and re-probed; otherwise, membranes from the same blot were split according to the molecular standard and then used to detect several proteins. The specific proteins were detected with a commercial enhanced luminol-based chemiluminescent (ECL) kit (Amersham). Densitometric analysis of bands was performed using Quantity One (Bio Rad).

### Caspase-1 activity and Interleukin-1 β analysis

Caspase-1 activity was measured in monocytes and neutrophils using a fluorometric caspase-1 assay (R&D systems) [25]. The fluorescence was recorded at 535 nm after excitation at 450 nm in a Chameleon multi-label detection platform (Hidex OY).

IL-1β concentrations were assessed in Heparin-plasma or in the supernatants of stimulated monocytes using a LINCOplex kit (LINCO Research Inc.) [25]. The samples were examined using Luminex® 100™ system. The data was analyzed using the software program StarStation 2.0 (Applied Cytometry Systems).

### Determination of reactive oxygen species (ROS)-production

A luminol-amplified chemoluminescence assay was used to determine production of ROS in isolated neutrophils stimulated with fMet-Leu-Phe (mostly an extracellular production) or PMA (mostly an intra-cellular production), as previously described [29]. The measurements were performed using a six-channel Biolumat LB9505 (Berthold Co., Wildbad, Germany).

### CD11/CD18-integrin expression and affinity

Integrins expressed on cells in whole blood were immuno-labelled [31] with fluorescence-conjugated antibodies either recognizing the CD11b/CD18-integrin or the ligand-binding domain of the CD11b-integrin (CBRM1/5) [32]. Contaminating erythrocytes were lysed, the samples were centrifuged, and the cells fixed in 0.1% paraformaldehyde (PFA). Granulocytes (predominantly neutrophils) were gated using FSC versus SSC, and mean fluorescence intensity of 5–10 000 cells/sample was subsequently analysed by flow cytometry (FACSCalibur). Nonspecific binding was determined using an isotypic antibody, and autofluorescence was routinely analysed, and found to be negligible.

### Cytokine production by macrophages stimulated with apoptotic neutrophils

Infected or uninfected neutrophils were washed three times in PBS containing 50 µg/mL gentamicin to clear extracellular bacteria, resuspended in DMEM without serum and added to macrophages (5×10^5^ neutrophils/well) at 2∶1 (neutrophil∶macrophages) ratio, and the supernatants were collected after 20 h. As a control, apoptotic neutrophils alone were incubated for 20 h in DMEM without serum. The supernatants were centrifuged to remove particular debris and stored in aliquots at -70°C. Cytokine concentrations in the culture supernatants were detected by ELISA, using ELISA MAX™ SET *Deluxe* kits according to the instructions provided by the manufacturer Nordic BioSite (Täby, Sweden).

To assess the number of ingested apoptotic neutrophils in macrophages, we performed a phagocytic assay using Molecular Probes CellTracker™ Green CMFDA (5-chloromethylfluorescein diacetate) for labelling of neutrophils prior to exposure to bacteria or UV irradiation (as control of non-infected apoptotic cells). Macrophages were allowed to interact with the apoptotic cells no longer then 2 h since the fluorescence of apoptotic cells co-incubated and digested by macrophages for 20 h were low in fluorescence. After 2 h the wells were vigorously washed to remove non-ingested neutrophils and the macrophages were scraped with cold Krebs-Ringer glucose (KRG) and analysed by flow cytometry or fixed in 2% PFA in KRG for 20 min (for macrophages cultured on glass cover slips), mounted with ProLong® Gold anti-fade reagent (Molecular Probes) and analysed by microscopy.

### Ethical considerations

Genetic analyses, as well as the cell biology analyses were performed after written informed consent from patients and the control persons. The study protocol was approved by ethics committee at Linköping University, and the study was conducted in accordance with the ethical guidelines of Declaration of Helsinki.

### Statistical analysis

Statistical analysis was performed using Prism 4 for Macintosh (version 4.0a) from GraphPad Software (GraphPad, San Diego, CA). For the age and gender matched data, the paired two-tailed Student's t-test was used, unless otherwise indicated. *p* values<0.05 were considered statistically significant.

## Results

### Markers of inflammation

The patients, who were carriers of the combined *NLRP3* (Q705K) and *CARD-8* (C10X) polymorphisms, displayed increased inflammatory responses, with raised levels of C-reactive protein (CRP), elevated erythrocyte sedimentation rate (ESR) and white blood cell (WBC) count (Table 1). Also, the spontaneous caspase-1 activity (data not shown) as well as IL-1β levels in plasma and cell supernatants were elevated in these patients compared to their controls (Table 1).

### Spontaneous apoptosis of neutrophils from patients with polymorphisms in the NALP3 inflammasome

**Figure 1:**
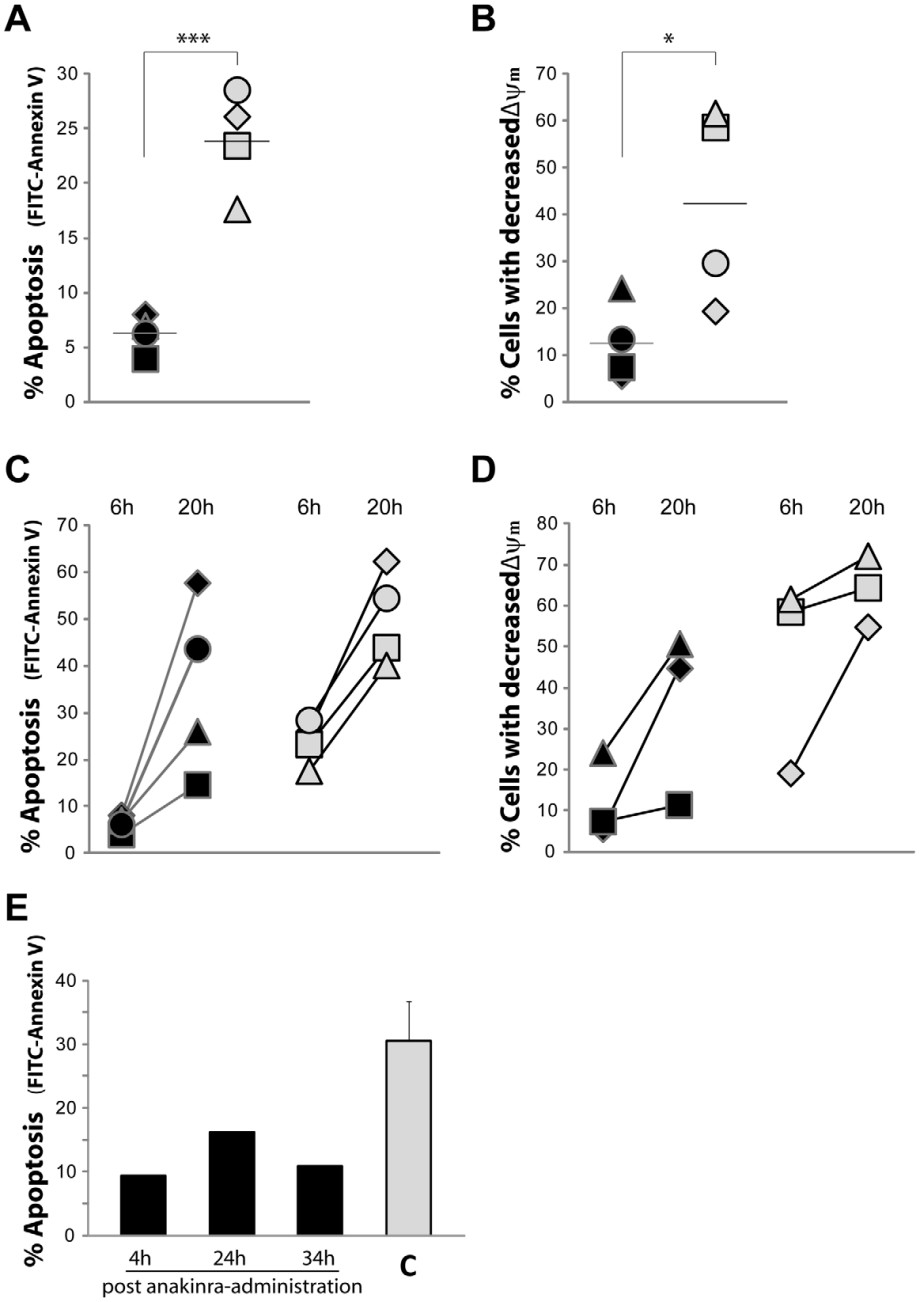
Spontaneous apoptosis of neutrophils from patients with polymorphisms in the NALP3 inflammasome. Neutrophils, isolated from whole blood of patients (black symbols/bars) or controls (C; grey symbols/bars), were incubated at 37°C for 6 h (**A**, **B**, **E**) or 20 h (**C**,**D**) after which the degree of apoptosis was evaluated by flow cytometry by detecting the surface exposure of phosphatidylserine using FITC-conjugated annexin V (**A**, **C**, **E**) and by assessing the mitochondrial membrane potential (ΔΨm) of the neutrophils by the use of TMRE (**B**, **D**). (**A–D**) Data are presented as % FITC-Annexin V+ cells or % TMRE-dim cells for each patient (P1♦, P2▪, P3▴, P4•) and its matched control (grey symbols). Black hyphens indicate mean values. (**E**) The degree of neutrophil apoptosis was investigated in one patient (P1) at 4, 24 and 34 h post anakinra-administration, and each bar show mean values from two separate experiment performed at different days for the patient (black bars) and mean values ± SD for 6 matched controls (C; grey bar). * and *** represents significant difference: *p*<0.05 and <0.001, respectively.

After 6 h of *in vitro* culture, the patients' neutrophils displayed significantly delayed spontaneous apoptosis (mean 6.2%) compared to neutrophils from healthy controls (mean 23.8%; Figure 1A), as detected by annexin V-stained phosphatidylserine. During apoptosis, mitochondrial damage is part of the apoptotic pathway via activation of caspases, which in turn results in decreased mitochondrial membrane potential (ΔΨm). By using the cationic and lipophilic dye TMRE, mitochondrial damage was evaluated by flow cytometry as percentage of cells with a decreased ΔΨm. After 6 h of *in vitro* culture, the patients had fewer cells with decreased ΔΨm compared to the controls (12.4% versus 42.1%; Figure 1B).

By 20 h of *in vitro* culturing, an overall increased spontaneous apoptosis was evident, but was still delayed in patients (mean 35.5%) compared to the control (mean 50.2%; Figure 1C). Also, the number of neutrophils with decreased ΔΨm was reduced among patients compared to controls after 20 h (35.5 vs. 63.7%; Figure 1D). Taken together, these data showed that neutrophils from patients with polymorphisms in the NALP3 inflammasome exhibit delayed apoptosis.

To test whether IL-1β blockade influence the rate of neutrophil apoptosis, blood from one of the patients (P1) was sampled at different time-points after administration of the IL-1 receptor antagonist anakinra. Delayed apoptosis was observed in the patient's neutrophils regardless if tested 4 h, 24 h or 34 h after administration of the drug (Figure 1E). To reduce pain and suffering for the patients, all subsequent experiments were therefore performed ≥24 h after administration of the drug (unless otherwise stated).

### Expression of anti-apoptotic protein Mcl-1 and Akt in neutrophils

**Figure 2:**
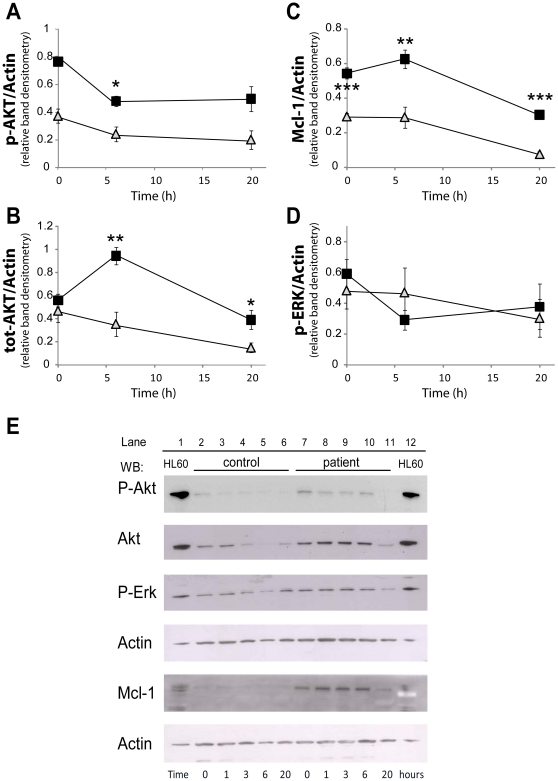
Expression of anti-apoptotic protein Mcl-1, Akt and ERK in neutrophils. Neutrophils, isolated from whole blood of patients (P1–3; black squares) or controls (grey triangles), were incubated at 37°C for indicated times, before lysed. Samples were analyzed by SDS-PAGE and immunoblotted with anti-bodies directed against phosphorylated AKT (**E**: first lane), total AKT (**E**: second lane), phosphorylated ERK (**E**: third lane), or Mcl-1 (**E**: fifth lane). The blots were stripped and re-probed with β-actin as a control for equal loading (**E**: fourth and sixth lane). (**E**) The blots shown are representative from 3 (patients) or 5 (controls) independent experiments. (**A–D**) The blots were scanned and the relative intensity of each band was quantified, and data are normalized to β-actin and presented as mean for two (patients: 1 and 3 h) or mean ± SEM for three (patients: 0, 6 and 20 h) or five (controls) independent experiments. *, ** and *** represents significant difference: *p*<0.05, <0.01 and <0.001, respectively.

The finding that neutrophils from patients with *NLRP3*(Q705K)/*CARD-8*(C10X) polymorphisms in the NALP3 inflammasome showed delayed apoptosis, prompted us to investigate how different regulators of apoptosis were involved in these patients. Neutrophils do not express the anti-apoptotic protein Bcl-2, but instead the homologue Mcl-1 is considered a major survival signalling protein. *De novo* synthesis and turnover of Mcl-1 is correlated with the reduction in apoptosis [33] and gradual loss of Mcl-1 during apoptosis releases pro-apoptotic Bcl-2 proteins from their Mcl-1 heterodimerization, which ultimately leads to mitochondrial membrane permeabilization and apoptosis [34]. Already directly after isolation, the neutrophil expression of Mcl-1 was 1.8-fold higher in patients compared to the healthy controls (*p*<0.001; Figure 2C and E). The expression of Mcl-1 in the patients' neutrophils increased during *in vitro* culturing, reaching 2.2-fold difference after 6 h (*p*<0.01) and declined at 20 h of incubation, but was still significantly higher than the maximum Mcl-1 expression in the controls' neutrophils seen at any time-point (Figure 2C and E).

The PI3K/Akt pathway relays its anti-apoptotic signals via phosphorylation of Bid and induction of Mcl-1 gene expression [35]. In the patients' neutrophils, both total and phosphorylated Akt were higher at all time-points measured (Figure 2A and B). Although, the level of phosphorylated Akt peaked at start (0 h) of *in vitro* culture for neutrophils from P1 and P3, and at 6 h for P2, the level of phosphorylation of Akt in the group as such (compiled data P1–P3; Figure 2A) was substantially higher and more sustained than in the five tested healthy controls (significant at 6 h, *p* = 0.021). At start of culture, the total Akt expression was almost similar in neutrophils from both patients and controls (Figure 2B and E). Whereas the total Akt expression gradually decreased during culture for control neutrophils, the expression of Akt in patients' neutrophils increased with time, and peaked at 6 h with 2.7-fold more Akt (*p* = 0.0079), and stayed significantly increased compared to controls also at 20 h (*p* = 0.024). The Mcl-1 and Akt data indicate sustained presence of survival signals in the neutrophils carrying Q705/C10X inflammasome polymorphisms.

Involvement of the anti-apoptotic protein ERK (p42/44 mitogen-activated protein kinase, p42/44MAPK) was also investigated (Fig. 2D and E). In neutrophils from both patients and controls, there was a sustained expression of total ERK protein during the course of *in vitro* culture (data not shown). In addition, there were no significant differences in neither total nor phosphorylated ERK between neutrophils from patients and controls.

### The effect of Akt and ERK inhibition on delayed apoptosis

**Figure 3:**
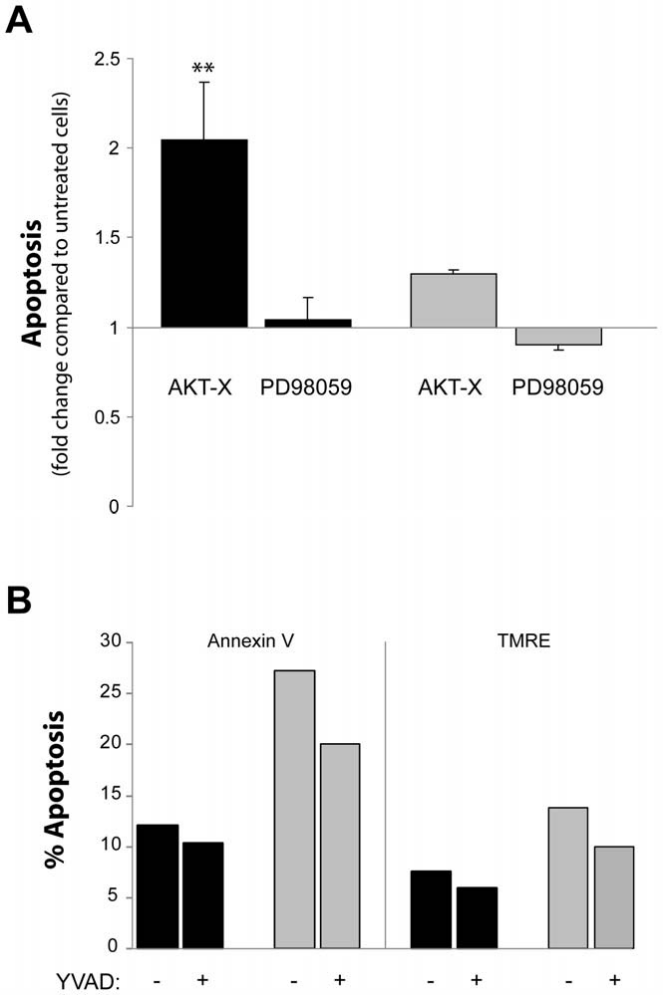
The effect of Akt and Erk inhibition on the delayed apoptosis of neutrophils. Neutrophils, isolated from whole blood of (**A**) 3 patients (P1–2, P4; black bars) or 5 controls (grey bars), or (**B**) from whole blood of P1 (black bars) and its matched control (grey bars), were incubated at 37°C for 6 h with or without inhibitors (AKT-X = Akt inhibitor, PD98059 = ERK inhibitor, z-YVAD-fmk = caspase-1 inhibitor). The degree of apoptosis was evaluated after 6 h by flow cytometry by detecting the surface exposure of phosphatidylserine using FITC-conjugated annexin V (**A,B**), and by assessing the mitochondrial membrane potential (ΔΨm) of the neutrophils by the use of TMRE (**B**). The data shown represent (**A**) the mean values from three (black bars) or five (grey bars) individual experiments ± SEM expressed as fold of untreated cells, or (**B**) the mean of two individual experiments. Student's t-test was used to compare each patient's neutrophils with vs. without inhibitor (** represents significant difference: *p*<0.01).

It has previously been shown that inhibition of Akt enhances apoptosis [36]. To further link together the delayed apoptosis in the patients with an up-regulated expression and activation of the anti-apoptotic proteins, we investigated the effect of Akt/ERK inhibition in regulating apoptosis. Isolated neutrophils were incubated with the Akt or ERK inhibitors, AKT-X and PD980559, respectively. Inhibition of Akt, but not of ERK, significantly enhanced apoptosis in the patents' neutrophils (Figure 3A) to an apoptotic level similar to untreated cells from the control individuals (39±7.2 vs. 42±2.3% apoptosis), whereas inhibition of Akt resulted only in minimal effects on spontaneous induced cell death in the control cells (Figure 3A). The role of Mcl-1 could not be further investigated, since there are no available pharmacological inhibitors against Mcl-1.

### Inhibition of caspase-1

To analyze whether the enhanced caspase-1 activity is directly linked to the delayed apoptosis in the patients, we utilized the caspase-1 inhibitor z-YVAD-fmk. Apoptosis was not restored in the cells from the patients after exposure to the inhibitor. Instead, neutrophils obtained from both patients and control subjects showed slightly decreased apoptosis and mitochondrial damage (Figure 3B).

### Priming and expression of β2-integrins

It has been shown that inflammatory and exudate neutrophils are primed and show enhanced expression of ß2-integrins [37] and delayed apoptosis [14]. To evaluate if the neutrophils obtained from patients were pre-activated, the expression and affinity of the β2-integrin CD11b/CD18 was analyzed. No activation of CD11b/CD18 was discernible when detecting the total amount of CD11b/CD18-integrins, as well as the number of high affinity CD11b-integrins, suggesting that the observed effects were not due to priming (Table 1). The expression and affinity of CD11b/CD18-integrins were similar regardless if measured 4 h, 24 h or 34 h after anakinra administration (data not shown).

### Generation of reactive oxygen radicals (ROS)

Since the generation of ROS is suggested to play an important role in apoptosis of neutrophils and macrophages [38], [39], [40], [41], we assessed the amounts of ROS after challenging with the bacterial peptide fMet-Leu-Phe or with the phorbol ester PMA that induces a receptor-independent response. No significant differences were detected between the patients' and controls' neutrophils neither of fMet-Leu-Phe nor PMA generated ROS (Table 1).

### Spontaneous apoptosis in asymptomatic carriers vs. non-carriers

**Figure 4:**
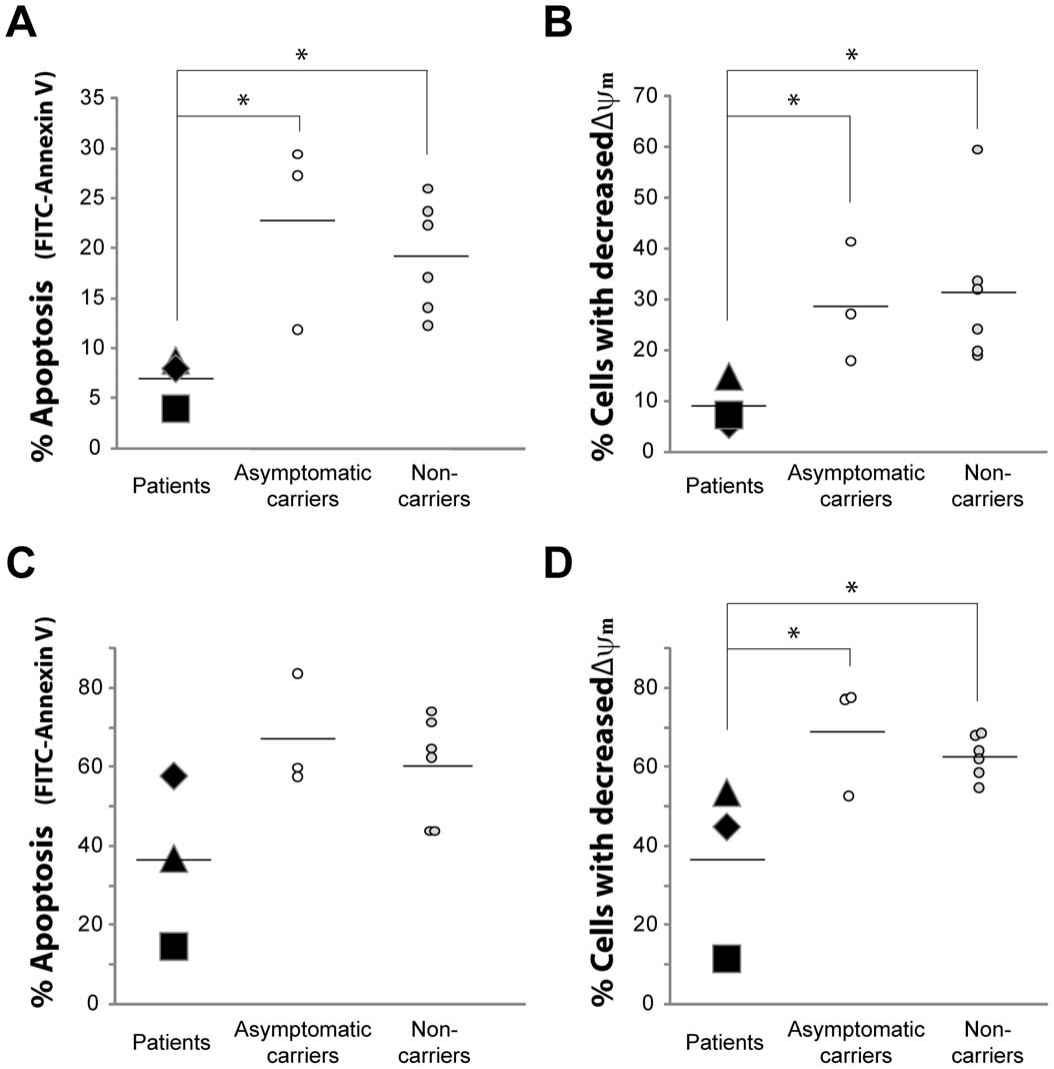
Spontaneous apoptosis of neutrophils from patients, asymptomatic carriers and non-carriers. Neutrophils, isolated from whole blood of patients (P1♦, P2▪, P3▴) and asymptomatic carriers of the *NLRP3* (Q705K)/*CARD-8* (C10X) polymorphisms and of non-carriers, were incubated at 37°C for 6 h (**A**,**B**) and 20 h (**C**,**D**). The degree of apoptosis was evaluated (10 000 cells/sample) by flow cytometry by detecting the surface exposure of phosphatidylserine using FITC-conjugated annexin V (**A**,**C**) or by assessing the mitochondrial membrane potential (ΔΨm) of the neutrophils by the use of TMRE (**B**,**D**). Data are expressed as % FITC-AnnexinV positive cells or as % cells with decreased membrane potential. Black hyphens indicate mean values for n = 3 (patients and asymptomatic carriers), or n = 6 (non-carriers) run in 3 separate experiments performed in triplicates. One-way ANOVA test with Bonferoni post test was used to determine significance between the different groups (* represents *p*<0.05).

Given the fact that the *NLRP3* (Q705K)/*CARD-8* (C10X) polymorphisms are common in the Swedish population; we wanted to elucidate if their presence *per se* affect apoptosis. Three asymptomatic carriers of the combined polymorphisms and 6 non-carriers, collected from healthy blood donors, were examined. There were no differences in IL-1β levels in plasma between asymptomatic carriers and non-carriers (0.41±0.04 vs. 0.43±0.18 pg/mL). In addition, spontaneous apoptosis, analyzed by both annexin V and ΔΨm, did not significantly differ between the carriers and non-carriers, neither after 6 h nor 20 h (Figure 4); suggesting that the polymorphisms *per se* are not enough for disease development.

### Microbe-induced apoptosis

**Figure 5:**
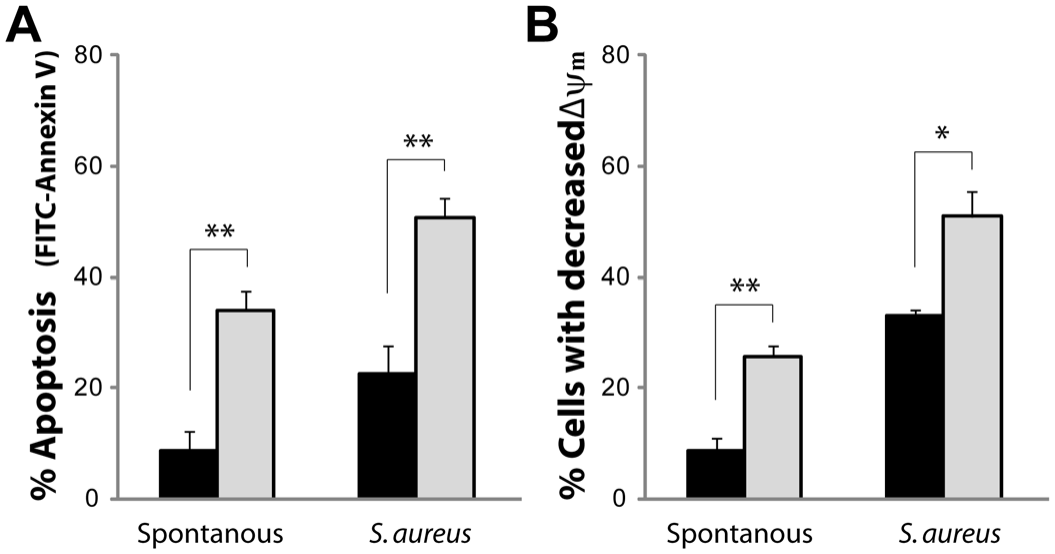
Microbe-induced apoptosis of neutrophils from patients with polymorphisms in the NALP3 inflammasome. Neutrophils, isolated from whole blood of 3 patients (P1–3; black bars) or 3 controls (grey bars), were incubated with *S. aureus* (bacteria∶cell ratio of 10∶1) at 37°C. After 6 h, the degree of apoptosis was evaluated by flow cytometry (**A**) by detecting the surface exposure of phosphatidylserine using FITC-conjugated annexin V, and (**B**) by assessing the mitochondrial membrane potential (ΔΨm) of the neutrophils by the use of TMRE. Data shown are % FITC-Annexin V+ cells and % TMRE-dim cells, respectively, presented as mean values from three individual experiments ± SEM. * and ** represents significant difference: *p*<0.05 and <0.01, respectively.

Next, we investigated the impact of microbes on neutrophil apoptosis using *S. aureus* as a microbial stimulus. The level of microbe-induced apoptosis, analyzed by both annexin V and ΔΨm, after 6 h was enhanced compared to spontaneous apoptosis in neutrophils from both patients and control subjects (Figure 5A and B). Even so, the microbe-induced apoptosis was still delayed in neutrophils isolated from the patients compared to cells from the control subjects (Figure 5A and B).

### Secretion of TNF in macrophages stimulated with apoptotic neutrophils

**Figure 6:**
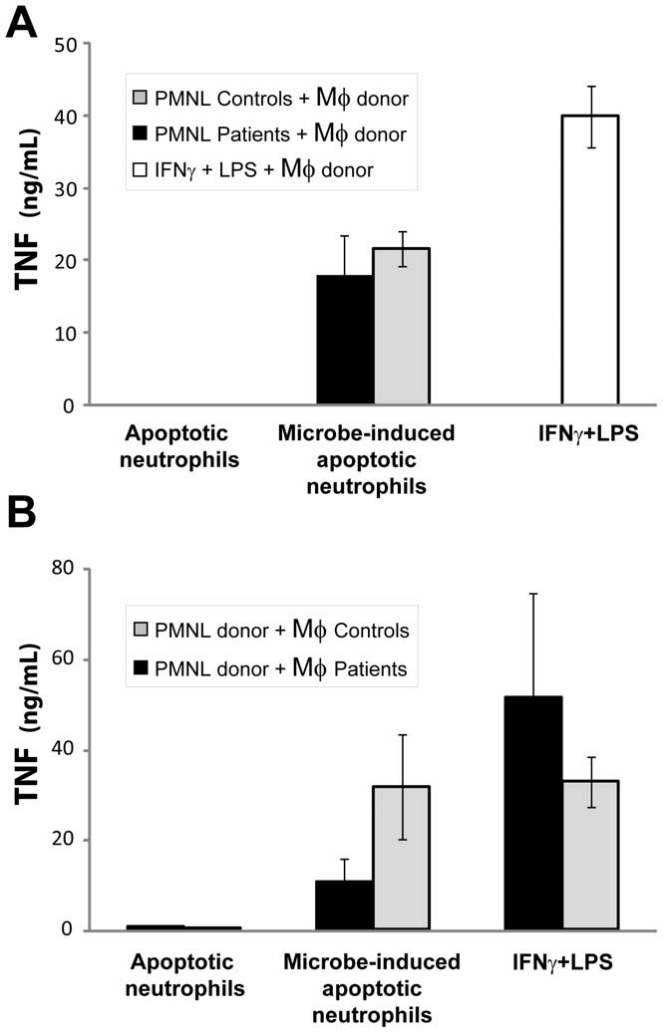
TNF production by macrophages stimulated with apoptotic neutrophils. (**A**) Neutrophils (PMNL), isolated from whole blood of a patient (P1; black bars) or 3 controls (grey bars), were left untreated (spontaneous apoptosis) or treated with *S. aureus* (microbe-induced apoptotic neutrophils) and given to donor blood-derived macrophages (Mφ). (**B**) Neutrophils, isolated from whole blood of blood donors, were left untreated (spontaneous apoptosis) or treated with *S. aureus* (microbe-induced apoptotic neutrophils) and given to blood-derived macrophages from patients (P1–2; black bars) or 4 controls (grey bars). (**A**,**B**) Macrophage supernatants were collected after 20 h, and TNF concentrations were detected by ELISA. Macrophages stimulated with IFNγ and LPS were used as positive controls. The bars represent mean ± SEM for three independent experiments.

Macrophages play an important role in clearing the tissue from apoptotic cells and thereby regulate resolution of inflammation. We have previously shown that when infected apoptotic neutrophils are cleared by macrophages, a pro-inflammatory response is elicited with release of pro-inflammatory cytokines, like TNF and IL-12 [28], [42]. In the present patients, the enhanced accumulation of neutrophils may put a great challenge on the clearing capacity of tissue macrophages. Impaired capacity to clear apoptotic cells could thus affect both resolution of inflammation and tissue damage during infection. First, the pro-inflammatory triggering capacity by infected and non-infected apoptotic neutrophils from patients was compared to those from controls. Neutrophils from patients or controls were treated with *S. aureus* to allow phagocytosis and induction of apoptosis [28], and then exposed to macrophages derived from monocytes isolated from blood of health blood donors. Following 20 h of co-culture, macrophage-secreted TNF was measured in the culture supernatants. There was no difference between patients' or controls' neutrophils in their ability to induce macrophages to produce the pro-inflammatory cytokine TNF (Figure 6A). However, the macrophages response was elicited only with bacteria-induced apoptotic neutrophils.

Second, we investigated if there was a difference in the ability of patients' and controls' macrophages to respond to infected and non-infected apoptotic neutrophils, in order to discern differences in activation and clearance. Donor blood-derived neutrophils were used to stimulate either control or patient derived macrophages. When evaluating TNF produced (Figure 6B), the patients' macrophages showed decreased production of TNF in response to bacteria-induced apoptotic neutrophils. Still, although the patients' macrophages showed a 50–65% decreased ability to produce TNF in response to bacteria-induced apoptotic neutrophils compared to control macrophages, there was no difference in TNF in patients and controls when stimulated with IFNγ+ LPS. To rule out the possibility that the observed effect was due to reduced capacity of the patient macrophages to phagocytose, we assessed, by microscopy, the number of apoptotic neutrophils taken up by the macrophages. Quantification of the number of apoptotic cells taken up by the macrophages showed that there was no significant difference in phagocytosis capacity between patient and control macrophages (data not shown).

## Discussion

To regulate the acute inflammatory process, a balance must exist between accumulation and elimination of exudate inflammatory cells, primarily neutrophils. This process is regulated by spontaneous or microbe-induced apoptosis, together with the subsequent elimination of apoptotic cells by tissue macrophages [16], [43]. Defects in apoptosis and/or elimination of neutrophils may lead to profound and devastating inflammatory and immunological conditions. Since a hallmark of the patients in the present study is leukocytosis, we raised the question whether the inflammatory feature was related to delayed apoptosis of neutrophils and/or impaired responses to clearance of neutrophils by tissue macrophages.

For all four patients, who were carriers of the combined polymorphisms of *NLRP3*(Q705K)/*CARD-8*(C10X) and showing an inflammatory disease phenotype, a delayed spontaneous as well as microbe-induced apoptosis was clearly observed. It is known that inflammatory and exudate neutrophils display delayed apoptosis [44], [45]; a mechanism which could explain why the patients' cells showed altered apoptosis. However, the delayed apoptosis was apparent irrespective of clinical symptoms, since anakinra treatment, which effectively reversed inflammation, did not affect the apoptotic process. Although there is no correlation to clinical symptoms, one could anticipate that the patients' neutrophils where primed due to constitutive IL-1β producing activity. Nevertheless, the patients' neutrophils showed no signs of priming or activation as demonstrated by an equivalent expression of CD11b/CD18 as compared to controls. These results rule out an enhanced anti-apoptotic effect caused by CD11b/CD18-integrins, known to occur during adhesion and transmigration [46].

One major pathway involved in neutrophil apoptosis is linked to the production of ROS, generated by the membrane-bound NADPH-oxidase [38], [47], [48]. ROS may damage lysosomes, leading to release of cathepsins, which in turn triggers the release of cytochrome C from mitochondria ultimately affecting its membrane potential [27], [49], [50]. We could not detect any altered ROS production in the patients' neutrophils. However, there was still a difference in mitochondrial damage between patients and controls, suggesting that the intrinsic pathway was affected in a ROS-independent way. This interpretation is further strengthened by the fact that the expression levels of the anti-apoptotic protein Mcl-1, which normally protects the mitochondria, is increased in the patients' neutrophils. Lacking both Bcl-2 and Bcl-X, and showing only sub-detectable levels of A1, neutrophils rely on Mcl-1 to regulate apoptosis. Mcl-1 levels are dynamically regulated by activated transcription and translation, but also by changes in its targeting to the proteasome [51]. It has been shown that activation of PI3K/Akt and ERK induce posttranslational modification of Mcl-1, thereby increasing its stability during GM-CSF signalling of neutrophil survival [21]. Our observation that both Mcl-1 and Akt are increased suggests that the key feature during the delayed apoptosis in the patients' neutrophils could be impaired Mcl-1 turnover. Whether this is due to increased GM-CSF activity and an overall decreased neutrophil cell death is, however, not clear.

Inflammatory conditions with increased IL-1β, display increased levels of Mcl-1 and delayed neutrophil apoptosis [52], [53], [54]. The constant presence of high levels of IL-1β could thus be the trigger for elevated Mcl-1 levels and subsequent delayed apoptosis of the patient's neutrophils. However, blockade of IL-1β with anakinra, 4 h, 24 h, or 36 h prior to neutrophil isolation, did not restore the delayed constitutive apoptosis; data arguing against IL-1β as the main mediator for the elevated Mcl-1 activity and reduced apoptosis in the patients carrying the *NLRP3* (Q705K)/*CARD-8* (C10X) polymorphisms.

Based on our previous results from monocytes isolated from one patient [25], as well as data from the present study, and from studies with transduced THP-1 cells (manuscript submitted), the *NLRP3* (Q705K) is a gain-of-function polymorphism giving rise to a constantly active inflammasome with constitutive IL-1β production; in a similar way as found for CAPS mutations [4], [19]. Blocking caspase-1 did however not restore the delayed apoptosis in the patients' neutrophils; data that may question a connection between the altered apoptosis and SNPs of the NALP3-inflammasome. Monocytes carrying CAPS-associated *NLRP3* mutations rapidly undergo cell death involving loss of mitochondrial inner transmembrane potential [23], [24]. This cell death is also found to be independent of caspase-1. Still, the ability to induce cell death is interconnected to the disease-associated mutations in *NLRP3*, as demonstrated by investigating a patient with *NLRP3* mosaicism carrying both mutant and non-mutant cells [24]. In addition, monocyte cell death in CAPS-patients was found to be dependent on cathepsin B [24]. Whether the delayed neutrophil apoptosis found in our patients is regulated by cathepsin B or not is not elucidated in the present study, but it has been reported that zVAD-fmk and other caspase inhibitors are also potent cathepsin B inhibitors [55]. This possibility can therefore not be ruled out. Taken together, these data show that the contribution of caspase-1 and IL-1β may be of less importance in *NLRP3*-induced cell death.

In agreement with our results, Saito *et al.* [24] found that the altered monocyte cell death in CAPS patients was not mediated by the inflammatory milieu and was independent of IL-1-targeted therapy. In addition, the cell death was independent of disease severity, and a small number of monocytes carrying *NLRP3* mutations were sufficient to evoke systemic inflammation in CAPS mosaic patients. In our study, the patients, albeit constituting a heterogeneous group, were all admitted to the clinic due to inflammatory related diseases without specified diagnosis. They were found to be carriers of the *NLRP3* (Q705K)/*CARD-8* (C10X) polymorphisms, displaying neutrophils with altered apoptosis that was apparent irrespective of clinical symptoms. To understand the impact of a dysfunctional NALP3-inflammasome in disease and to clarify the mechanism of neutrophil cell death in these patients, additional experiments are needed. Also, we cannot exclude the possibility that there are other types of genetic abnormalities that may influence the inflammatory disorders of these patients.

Q705K in combination with C10X has been implicated in diseases like Crohn's disease, rheumatoid arthritis and aortic aneurisms [56], [57], [58], [59]. The fact that *NLRP3* (Q705K)/*CARD-8* (C10X) polymorphisms are quite prevalent in the healthy population, taken together with the heterogeneity of these diseases, as well as of the symptoms of the patients of this study, suggests these polymorphisms to act as susceptibility factors that in conjunction with an environmental cue, such as an infection or by additional genetic variations, predispose for enhanced inflammation. This hypothesis is supported by the present study showing similar inflammasome status, measured as degree of apoptotic neutrophils and production of IL-1β, between asymptomatic carriers and non-carriers. Also, data from transduced THP-1 cells indicate Q705K/C10X as gain-of-function polymorphisms of low-penetrance (manuscript submitted) that may thereby only participate in an inflammatory phenotype depending on other host related genetic and/or environmental factors. Environmental factors are suggested to influence disease severity for polymorphism involved in familial Mediterranean fever [60], [61].

The NALP3 inflammasome is believed to function as an intracellular pattern-recognition receptor for gram-positive (G^+^) bacteria like *Staphylococcus aureus* [6]. In *ex vivo* studies of healthy individuals, we have previously found a 3-fold increase in IL-1β upon challenge with *S. aureus* [25]. In a patient with the *NLRP3* (Q705K)/*CARD-8* (C10X) polymorphisms, IL-1β was found to increase 28 times after incubation with this microbe [25]. In the present study, both patients and control subjects showed an augmented microbe-induced apoptosis as compared to spontaneous apoptosis. Several microbial components (*e.g.* LPS, peptidoglycan and CpG DNA) may all rescue neutrophils from apoptosis [15]. Even so, the *S. aureus*-induced apoptosis in neutrophils isolated from the patients was significantly delayed compared to cells from the control subjects. The finding that microbe-induced apoptosis is significantly impaired in the patients, indicate that the increased activity of Mcl-1 and Akt not only affected the spontaneous apoptosis, but also protects the cell from a strong exogenous apoptotic stimulus.

A main feature of tissue macrophages during resolution of inflammation is to phagocytose apoptotic neutrophils, not only after constitutive apoptosis but also apoptosis that follows ingestion and killing of invading pathogens. We have earlier shown that pathogen-induced apoptotic neutrophils trigger a pro-inflammatory response in macrophages [28], [42], suggesting that apoptotic neutrophils can enhance the early innate and the adaptive host response to certain pathogens. Lack of macrophage response to apoptotic cells could lead to sustained inflammation and impaired clearance of microbes. The fact that macrophages from patients with the *NLRP3* (Q705K)/*CARD-8* (C10X) polymorphisms responded weakly to pathogen-induced apoptotic neutrophils show that the link between the early innate and the adaptive immune response is impaired in these patients. If this has any impact on their susceptibility to specific pathogens remains to be investigated.

To conclude, we have shown that not only the macrophage response is altered in patients with the combined polymorphisms of the NALP3 inflammasome, but also the constitutive and pathogen-induced apoptosis of neutrophils. Whether this is due to the effect(s) of extracellular anti-apoptotic factors in plasma or altered intrinsic regulatory mechanisms, is still unclear. The impaired turnover and clearance of apoptotic neutrophils could however be one phenotypic characteristic affecting the clinical feature of these patients both during immunological homeostasis and when encountering different exogenous stimuli.

## Supporting Information

Data S1 Case Reports.
(DOCX)
