# Supplementary material for: Common Genetic Variations in the NALP3 Inflammasome Are Associated with Delayed Apoptosis of Human Neutrophils
Source: PLoS One. 2012 Mar 5;7(3):e31326. doi: 10.1371/journal.pone.0031326 (PMC3293864; doi:10.1371/journal.pone.0031326)
Supplement: Data S1 — Case Reports. (DOCX) [file pone.0031326.s001.docx]

***Case reports***

***Patient 1***

This male patient, born in 1977, has previously been presented [[1](#_ENREF_1)]. To recapitulate: Gonarthritis first appeared at the age of 8 following a *Streptococcus* infection. HLA-B27 was positive, and the patient’s father suffered from ankylosing spondylitis. Large-joint arthritis has since then recurred several times and occasionally, muscle pain has dominated. During recent years, arthritis in large joints and persistent fever with constantly elevated CRP has dominated. Characteristic signs of Cryopyrin-Associated Periodic Syndromes (CAPS), such as urticaria, bony overgrowth of joints, aseptic meningitis and other CNS manifestations, chronic papilledema, sensorineural hearing loss, and short stature, were absent.

The patient has been extensively investigated several times but without definitive diagnosis. CT of sacroiliac joints has shown bilateral sacroiliitis grade II. ANA, ANCA, RF have been negative and complements normal. In May 2005, ferritin was significantly elevated (1572 μg/L) with corresponding findings of elevated CRP (117 mg/L), ESR (80 mm/h), and WBC (27 x10^9^/L; patient’s maximum value 89 x10^9^/L). The bone marrow picture has been reactive. All infectious investigations have been normal. Unspecified spondylarthritis was diagnosed according to the criteria of Amor [[2](#_ENREF_2)]. The New York criteria of ankylosing spondylitis were not fulfilled, as he never had any back pain or spinal stiffness. Still’s disease was considered, but excluded by other rheumatic diseases according to the Yamaguchi criteria [[3](#_ENREF_3)].

Over the years, azathioprine, sulfasalazine, methotrexate, cyclosporine A, colchicine, intravenous administration of immunoglobulin, moderate doses of cortisone, and NSAIDs was ineffective. Infliximab produced side effects, while the effects of adalimumab and etanercept have been inadequate. Immediately before the start of anakinra treatment, the patient was taking methylprednisolone (12 mg/day), methotrexate (12.5 mg/week), celecoxib (400 mg/day) and etanercept (25 mg/ twice a week). In early August 2005, treatment with anakinra (100 mg s.c. daily) was initiated. Within 24 hours, the patient was afebrile and free of arthritis. CRP dropped from 129 to 31 mg/L and WBC from 23.3 x10^9^/L to 10.9 x10^9^/L within 48 hours. These parameters became entirely normal within 1 week. Except for seborrheic dermatitis, no severe side effects have occurred. Today, anakinra is administrated daily, and no other anti-inflammatory drugs are given.

***Patient 2***

This male patient, born in 1952, was suffering from achalasia since 1995. The oesophagus was very wide and he got chronic nutrition via a percutaneous gastrotomy. In 2000, a symmetrical progressive peripheral polyarthritis including hands, feet, elbows, shoulders, and knees presented. Rheumatoid factor (RF) and anti-CCP were negative, whereas HLA-B27 was positive. No fever or back pain was present.

The response to methotrexate (20 mg/week), sulfasalazine (2000 mg/day) and prednisolone (10 mg/day) was not satisfying so infliximab was introduced in 2006 resulting in temporary improvement. Switch to etanercept and subsequently adalimumab was unsuccessful, both regarding clinical efficacy and laboratory tests (CRP maximum 210 mg/L, ESR 110 mm). X-ray of the hands and feet showed only 2 minimal erosions. In October 2007, anakinra was introduced (100 mg s.c. daily) in addition to subcutaneous methotrexate (25 mg/week) and prednisolone (5 mg/day). The patient improved but still had polyarthritis.

***Patient 3***

This female patient born in 1970, with a family history of systemic lupus erythematosus, was referred to the Rheumatology unit with morning fever, severe generalized myalgia and scarring acne. In her youth, examination at the Haematology unit due to elevated WBC did not reveal any underlying pathology. On examination by a rheumatologist in November 2007, a 5 x 4 cm irregular ulcerating skin lesion with surrounding erythema was observed on her right lower leg. In addition, she had exudative arthritis in the knees and wrists. Laboratory tests revealed elevated CRP (67 mg/L), ESR (88 mm/h) and WBC (17 x 10^9^/L; neutrophils count 14 x 10^9^/L). Liver tests, serum creatinine, urinalysis, and infectious tests including tuberculin test (PPD) were normal. HLA-B27, ANA, ENA, ANCA, RF, anti-CCP and cardiolipin antibodies were negative; complement factors C3 and C4 were normal. No malignancy was detected at abdominal and thoracic CT scanning.

A deep biopsy from the skin lesion was taken with histopathological findings compatible with pyoderma gangrenosum, but no signs of vasculitis were seen. Differential diagnoses, such as Behcet’s disease, Crohn’s disease and PAPA syndrome (pyogenic arthritis, pyoderma gangrenosum and acne) were considered. Colonoscopy was performed to exclude Crohn’s, but turned out completely normal. Furthermore, the patient was HLA-B51 negative, lacked oral and genital aphthous ulcerations and showed no signs of erythema nodosum or pathergy. Thus, the plausible working diagnosis was PAPA syndrome.

Initial treatment with prednisolone (30 mg daily) was successful in terms of prompt disappearance of fever, myalgia and arthritis as well as improvement of the skin lesions. ESR and CRP levels were almost immediately normalized. Intra-articular corticosteroid injections were also given in knees and wrists. When oral prednisolone was tapered below 15 mg daily in May 2008, generalised myalgia began to recur, and inflammation markers were again raised. The steroid dose was temporarily increased and colchicine (2 mg daily) was introduced. In combination with colchicine, the prednisolone dose could then gradually be tapered down to 5 mg daily (February 2009). Since then, the disease has remained essentially inactive without any skin lesions, and has, so far, not required additional therapy. Occasionally, however, the patient experiences myalgia and/or arthralgia with limited elevations of CRP and ESR.

***Patient 4***

This previously healthy man, born in 1944, presented in 2003 with generalized myalgia without swollen joints. CRP and other inflammatory parameters were increased. He was successfully treated with prednisolone (40 mg/day), but the symptoms and inflammation recurred when the dose was tapered (15 mg/day). Addition of methotrexate and later cyclosporin A, azathioprine, as well as cyclophosphamide were all unsuccessful.

In February 2007, morning fever and sweating presented. Inflammatory parameters were elevated: WBC (27 x 10^9^/L) with neutrophil granulocytes predominating, CRP (103 mg/L), and ESR (68 mm). Liver tests, serum creatinine, urine tests, and infectious tests including: PPD, ANA, ENA, ANCA, cardiolipin antibodies, complement C3 and C4, were all normal. Muscle biopsy revealed no myositis or vasculitis, and bone marrow aspiration appeared normal. Amyloidosis could not be detected in fat aspiration biopsy. No malignancy could be detected at abdominal and thoracic CT scanning but a small right-sided pleural effusion was found. Ultracardiography showed normal heart function.

Anakinra (100 mg s.c. daily) was started in May 2007, and the muscle pain and inflammatory parameters improved. However, 4 months later the muscle pain recurred during ongoing anakinra and prednisolone (20 mg/day). In December 2007 progressive dyspnea and oedema presented. A right-sided hemhorragic pleural effusion, liver enlargement and ascites developed. Constrictive pericarditis and amyloidosis were suspected but could not be verified. The patient died in August 2008 with severe heart failure, low blood pressure, continuously increased inflammatory parameters. Autopsy could not give a more definite diagnosis.

***Reference list***

1. Verma D, Lerm M, Blomgran Julinder R, Eriksson P, Soderkvist P, et al. (2008) Gene polymorphisms in the NALP3 inflammasome are associated with interleukin-1 production and severe inflammation: relation to common inflammatory diseases? Arthritis and rheumatism 58: 888-894.

2. Amor B, Dougados M, Mijiyawa M (1990) [Criteria of the classification of spondylarthropathies]. Revue du rhumatisme et des maladies osteo-articulaires 57: 85-89.

3. Yamaguchi M, Ohta A, Tsunematsu T, Kasukawa R, Mizushima Y, et al. (1992) Preliminary criteria for classification of adult Still's disease. The Journal of rheumatology 19: 424-430.
